# Endometriosis leading to frequent emergency department visits–women’s experiences and perspectives

**DOI:** 10.1371/journal.pone.0307680

**Published:** 2024-11-21

**Authors:** Christine Roman Emanuel, Herborg Holter, Ida Nygren Hansson, Maria Forslund

**Affiliations:** 1 Department of Anesthesiology, Operation and Intensive Care, Sahlgrenska University Hospital/Östra, Region Västra Götaland, Gothenburg, Sweden; 2 Institute of Health and Care Sciences at Sahlgrenska Academy, University of Gothenburg, Gothenburg, Sweden; 3 Department of Obstetrics and Gynecology, Institute of Health and Care Sciences, Sahlgrenska Academy, University of Gothenburg, Gothenburg, Sweden; 4 Department of Obstetrics and Gynecology, Sahlgrenska University Hospital, Gothenburg, Sweden; 5 Department of Obstetrics and Gynecology, Institute of Clinical Sciences, Sahlgrenska Academy, University of Gothenburg, Gothenburg, Sweden; Dipartimento di Scienze Mediche e Chirugiche (DIMEC), Orsola Hospital, ITALY

## Abstract

**Background:**

Endometriosis is a common condition affecting 1–10% of all women. The condition is highly associated with pain. Most women with endometriosis are treated as elective outpatients, but these patients sometimes need to visit the emergency department. The aim of this study was to describe experiences and expectations related to repeated gynaecological emergency (GED) visits among women with endometriosis.

**Method:**

This qualitative study with semi structured interviews were conducted with ten patients diagnosed with endometriosis who visited the GED at a tertiary university hospital four or more times within a 12-month period. Data were analysed by thematic content analysis.

**Result:**

Two main themes was identified which embodied the women’s overall experience, grouped into: “Living with pain” and “Patients´ needs when seeking GED”, with six underlying themes. Several women described their visit to the GED as a “lottery”, as they never knew what kind of treatment they would receive. Being listened to was most important for the women. Individualized care plans did not exist for most of the women in this study, although they repeatedly needed to visit the GED.

## Introduction

Endometriosis is a common condition in women, the prevalence of which has been reported up to 10% [1–3]. Endometriosis is characterized by the presence of endometrial cells outside the uterine cavity, which may cause irritation, inflammation, and scar tissue and result in symptoms such as dysmenorrhea, dyspareunia, and pelvic pain [1, 4, 5]. In many cases, the condition has a negative effect on women’s health-related quality of life and is associated with decreased emotional, physical, psychological, sexual, and social health [1, 6–9]. The “gold standard” for diagnosing endometriosis is a laparoscopy with histological confirmation of endometrial tissue. It often takes many years to get diagnosed and to find a proper treatment. During diagnosis, women meet many different healthcare professionals and diagnosis delay is associated with a lot of frustration. They frequently describe health professionals as problematic including normalization and trivialization of symptoms [10–14].

Most women with endometriosis are treated as elective outpatients, but sometimes they need to visit the emergency department (ED). Studies have shown that the high patient numbers at the ED often make it challenging to provide high-quality care, which is manifested, for instance, by delayed analgesics, causing undue suffering in patients with severe pain [15, 16] Although many different patients seek help at the ED, endometriosis is a common reason for seeking emergency care, especially at the gynaecological emergency department (GED) [11]. These patients often need help from a multidisciplinary team of health care professionals (HCPs). Despite this, little is known about patients with endometriosis from the ED perspective, and few studies have been conducted in the GED setting regardless of diagnosis. In addition, some patients, with or without endometriosis, repeatedly seek GED care, indicating unmet needs.

To increase knowledge about patients with endometriosis with repeat GED use, we performed the present qualitative study. The aim of this study was to increase knowledge about women with endometriosis repeatedly visiting the GED and investigate patient experiences and expectations.

## Methods

### Design

This qualitative interview study was performed at the GED of a university hospital in Sweden between October 2022 and January 2023. The GED has had approximately 15 000–16 000 visits per year, with an annual increase since 2017.

### Participants

Patients were eligible to participate if they had visited the GED four or more times within a 12-month period and had been diagnosed with endometriosis, according to the medical records during 1 jan 2021 to 1 jan 2022. Other inclusion criteria were the ability to communicate in Swedish and having no severe psychiatric diagnoses.

The patients were consecutively asked to participate based on the number of their visits to the GED during the previous year. Out of 17 women visited the GED four times or more, ten women with most visits within 12 months were first asked to participate after the inclusion criteria was fulfilled. The median number of times the women had visited the GED was eight (range four to 23). All initially invited women (n = 10) agreed to participate and signed informed consent before the interviews was conducted. The median age of the participants was 35 years (range 23–51 years). Details of their demographic characteristics are given in Table 1.

**Table 1:** Characteristics of the included participants, given as median (range) or percentage.

Age, yrs.	35 (23–51)
Age at start of symptoms, yrs.	13 (12–22)
Age when diagnosis was verified, yrs.	27 (21–34)
Time to verified diagnosis, yrs.	12 (2–21)
Time since endometriosis diagnosis, yrs.	6 (1–18)
Number of visits at the GED within 12 months	8 (4–23)
Number of visits at the GED before the inclusion period	11 (3–12)
Previous operations, n	1 (1–3)
Working full-time, %	70%
Highest education—high school	30%
—university	60%

### Data collection

The participants were initially sent digital information about the study. Approximately 1 week later, they were contacted by phone by two of the authors (C.R.E. and I.N.H.). At this time, they were given the opportunity to ask questions about the study and decide if they wanted to participate. When they gave their approval, an interview was scheduled, and a written informed consent was given before the interview started. The participants could choose if they preferred a face-to-face interview or a digital interview. Out of the total number of interviews conducted, eight were face to face and took place at the hospital and two were performed digitally. The median duration of the interviews was 44 (range 28–72) minutes, and the interviews needed to be rebooked a median of two (range one to seven) times. The interviews took place between 28 oct 2022 to 5 jan 2023.

All interviews were audio recorded and transcribed verbatim by a skilled transcriber outside the research team. All identifying details were removed.

The interviews followed a semi structured guide (see S1 File) focusing on experiences related to endometriosis and experiences related to visiting the GED. Questions included: How does endometriosis affect your life? Can you tell us about your experiences of health care in connection with endometriosis? How could endometriosis care be improved?

The interview guide was created by the research group, a multidisciplinary team. Two of the authors (C.R.E. and I.N.H.), both registered nurses but not working at the GED, performed the interviews. One of the interviews was conducted by the two authors together to align the approach.

### Data analysis

The interviews were analysed using thematic content analysis according to Braun and Clarke [17]. The data were coded using the data analysis software program NVivo (version 12). To familiarize themselves with the data, two of the authors (C.R.E. and H.H.) repeatedly read the transcribed data in its entirety, noting initial ideas. The entire data set was coded for interesting features. The coding was performed by the two authors (C.R.E. and H.H.) in duplicate, and differences in coding (which were only minor) were resolved by discussion to reach consensus. All codes were collated into preliminary themes; finally, after removing overlapping themes and merging similar ones, a master theme, two main themes, and six underlying themes were identified. A mind map was created to check that the themes covered the different codes.

Thereafter, the coded data and all transcripts were reread to ensure that the themes were representative of the data, and the main themes and subthemes were refined and revised by all authors until consensus was reached. A detailed analysis was conducted by the two authors C.R.E. and H.H., but the whole research team worked together through the analytical process to find the essence in the data and ensure that no important themes were missing.

### Ethical considerations

The study was approved by the Regional Ethical Review Board in Linköping, Sweden (2022-09-20, Dnr 2022-03306-01). All participants gave their oral and written informed consent to participate in the study.

## Results

**Fig 1:**
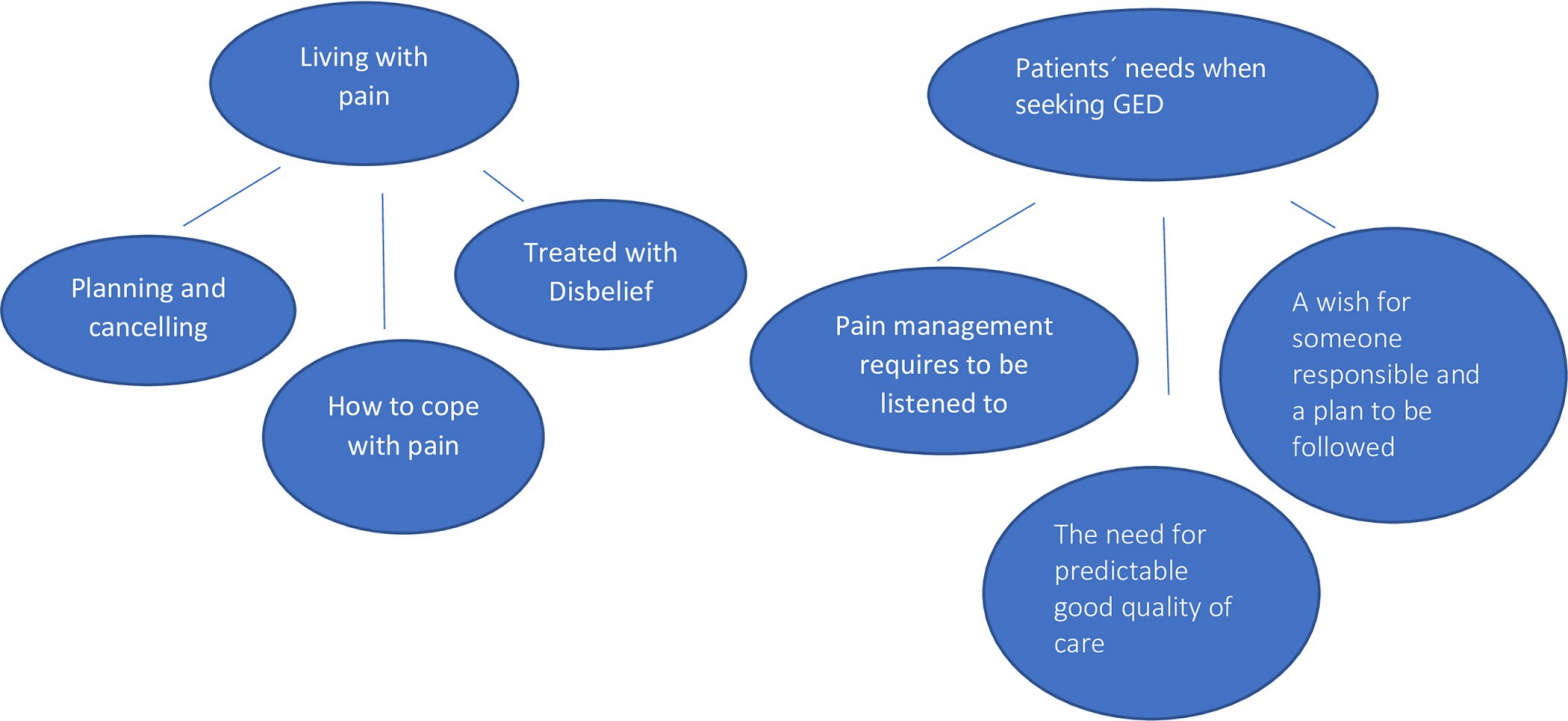
The analysis identified two main themes: ‘Living with pain’ and’ Patients’ needs when seeking GED´, including six underlying themes which is illustrated in Fig 1.

The analysis identified two main themes: “Living with pain” and “Patients’ needs when seeking GED”, including six underlying themes which is illustrated in Fig 1.

All themes are illustrated by quotations from the interviewed women and labelled with the interview number.

### Living with pain

*Well, I would certainly say that if I had not had endometriosis, I would indeed have… well but, like, lived. Because that’s what it feels like you’re not doing when you have endometriosis, that you’re not living, sort of. (1)*

#### Planning and cancelling

Not knowing when the pain was going to set in or how it would set in limited the women’s lives. The pain they experienced was often exhausting, and since it was unpredictable, it limited their social life and their ability to plan exercise routines. Several of the women said that exercise played a vital role in their social interactions. Therefore, when they were unable to engage in physical activity, they lost not only the benefits of activity themselves but also an important component of their social life.

*The first years with the pain were extremely hard. I was exhausted. I couldn’t take short walks, nothing. So everything like that was affected. (4)*

Unpredictable pain affected the women’s choice of activities. One woman described that when she planned to go to a concert, she had to know whether the chairs were comfortable to be able to manage. Some of the women described gratitude towards their friends who showed great understanding when they cancelled at short notice. Others related that they had no, or only a very limited circle of, friends because of repeated cancelling and their limited ability to plan activities.

*I’ve often had to cancel things at the last minute or been unable to say yes to things because I’ve had pain or haven’t had the energy because I was tired. (6)*

All the women described that it was not only the pain but also the tiredness and fatigue that affected their lives as well as what they could plan and what they were able to do. Women with a family or partner often experienced feelings of disappointment towards themselves at not being able to be the mother or partner they wished to be, on account of the chronic pain and fatigue. Pain and fatigue had a significant impact on their emotional wellbeing, leading to feelings of frustration and guilt, especially when they were unable to make plans or when they had to cancel an arrangement.

*It can affect your relationships as well, as I mentioned, because you just can’t be bothered. You don’t have, like, the same strength and energy to give to other people around you, and not everyone is, like, understanding of what it means to live with pain, and especially endometriosis pain. (9)*

The lack of ability to plan and the frequent need to cancel at the last minute affected the women’s lives at all levels, including their education and working life. During their time in school, many women had been absent due to pain. The women believed that if they had received their diagnosis earlier, it would have given them better opportunities in school, as a better understanding of their condition could have helped in providing a context for their absences and enabled more support.

*If I had got the diagnosis earlier or…, but it’s more like my schooling would have been different if I had not had endometriosis. I’ve missed out on some of my schooling and at work as it is. (8)*

Most of the women were employed and prioritized their work. They tried to live their lives as normally as possible. They found it challenging to take sick leave, as it was important for them to continue their professional commitments. Understanding from their manager and colleagues was crucially important.

*… always a very, very guilty conscience when I call in sick or if I need to go home halfway through the day because I’m having pain … (6)*

The women’s condition limited their opportunities for career development. Planning for career development was risky when understanding the working environment was so important for the women to manage their working life. Some women described that if they had not had endometriosis, they would probably have planned a new job, but since everything worked well with the work environment and manager, they felt it was safer to stay.

*… there’s safety, I have permission to adapt my job as I need to, which is truly great. (4)*

#### How to cope with pain

The knowledge and education the women received from HCPs about how the body reacts to pain was valuable to them. Education and knowledge about the pain helped the women cope with their pain on a daily basis. By learning how their body responded to pain, they were able to identify triggers and warning signs and develop strategies to manage their symptoms. This knowledge gave them a sense of control and empowered them to take an active role in their own health care.

*The most important thing for me was that we got to learn that coping with a daily life with pain is like coping with grief. Because so much of your life disappears … like, you can’t do what you’re accustomed to doing, when you have pain. (1)*

When the pain was moderate, the women used coping strategies such as painting, meditation, breathing practice, or going on walks or to a movie. Nonmedical treatments such as transcutaneous electric nerve stimulation (TENS), heating pads, and acupuncture were helpful for some of the women. They described these methods as crucial to stopping the pain from accelerating at an early stage. One woman expressed that the very acceptance of the condition and its effects on her body was a form of coping, making her come to terms with a life with endometriosis.

#### Treated with disbelief

Before being diagnosed with endometriosis, many women had an overall experience of disbelief and mistrust. Not being understood and acknowledged in their pain had affected their self-esteem.

‘*No, but this is just mental … –it’s all in your head, there’s nothing wrong with you.’ (5)*

The women felt that they could not live up to their own and others’ expectations and that they constantly disappointed everyone around them. The social environment frequently dismissed the severity of the pain, considering ‘a little pain’ to be ‘normal.’ As a result, many women came to believe that the pain and fatigue were a natural part of life.

*I think in general that it’s going around with a feeling of constantly letting people down. That’s certainly the overall feeling. (7)*

When the diagnosis of endometriosis was verified, most of the women had felt relief. They felt that the diagnosis proved them right. However, they also felt sadness. It became easier for them to understand their health condition; pieces of their life fell into place, and feeling vindicated strengthened their self-esteem.

*Then, it was like things fell into place a little, that it’s not me who is imagining things, but rather that it’s not normal to feel like I have, or like I did. (8)*

### Patients’ needs when seeking GED

*When you seek help, it’s at the GED. So then this fear clearly starts to grow within me, ‘Who is it that …?,’ ‘When should I go in?’ I try to drag myself in at the last minute and seek help, … until it’s intolerable, clearly.–Who wants to go there? No one. I go in and I’m thinking ‘I hope that it’s a good, understanding doctor … Kind, that’s all I’m asking for,’ one who treats me with respect and doesn’t sit with their back to me while typing on the computer. Just look me in the eye and talk to me like a fellow human being … (2)*

#### Pain management requires to be listened to

The primary reason for seeking care at the GED was to get help with pain management. Several of the women described how they waited a long time before seeking care at the GED. They wanted to manage the pain by themselves and did not want to seek help immediately, in the hope that the pain might lessen at home. As the pain intensified, they finally reached a point where they felt they had no other alternative than to seek help at the GED.

*No one thinks it’s fun to seek emergency help. It’s not something you do as a hobby, but you’ve tried all the aids you have at home before going in and … that it …, that you may not …, that they might not properly understand the strength it takes to venture out to get help and seek care. (8)*

The pain management depended on the HCP treating them. When the women perceived disbelief from the HCP, they usually received inferior pain management and felt diminished. Mistrust from the HCPs was described as more common before they had received their diagnosis, but it could also occur after the diagnosis, especially from some doctors.

When the GED was overcrowded, the women noticed that the HCPs were busy and sometimes stressed and that it was difficult to be heard and receive adequate help. Under these circumstances, and when they were uncertain about when the doctor would be able to see them, pain, stress and anxiety emerged, even if the women tried to accept the long waiting time to obtain an assessment.

*So then I find that it’s a bit annoying that it’s different …, depending on which nurse and which doctor you get, whether I get pain relief right away or whether I have to wait several hours for the doctor. I’ve been there for more than 16 hours sometimes. (4)*

The women often described the care they received from nurses as positive, as they were met with respect, listened to, and acknowledged in their pain. When the doctors were busy, the nurses often tried to meet the patients’ needs and ease their pain.

*I get to lie down, I get double heating pads, I get pain relief, like, whatever is needed depending on how bad my pain is … and they take me seriously, or at least it feels like they’re taking me seriously and they …, they understand in another way, it feels like. They show respect in a very, very vulnerable situation. (6)*

It was important to be listened to and to have their story acknowledged by the HCPs. When an HCP acknowledged a woman’s needs, the woman felt less stressed and more secure. Trust and respect in communication were essential for the whole visit.

*… actually listening to what the patient is saying. Now I’m not saying that we patients are always right … – … but we do often have truly, truly good …, or I can say in any case that I know my body very, very well, and when I say that ‘something isn’t as it should be’ I appreciate it if someone listens to me then. (4)*

#### The need for predictable good quality of care

The reason for waiting so long before seeking emergency help with pain management was also related to previous experiences at the GED. The care they received when seeking help was unpredictable. It could be good, if they were treated with care and respect; or it could be bad, when they were treated with ignorance and mistrust.

The women felt that seeking care at the GED was similar to playing the lottery, and a lot depended on which doctor they met.

*Why is it different depending on which doctor is on call? Why does it differ so much? What is it that causes it to differ so much? (2)*

The nurses and midwives were described as stable and secure, but they could also vary. The feeling of playing the lottery made the women hesitate to seek emergency care and was one of the reasons they stayed at home for longer than they should.

*And then I’m always super tense about which doctor I’m going to get. I’m always truly worried about that. Because the process is sort of like a lottery. (10)*

Seeing different doctors who were responsible for different medical issues, both at the hospital and in primary care, resulted in varying levels of care. The women expressed a need for better communication between health care institutions to improve their overall care experience and minimize the feeling of being at the mercy of chance.

Most women described that knowledge about endometriosis among HCPs and information about the condition generally had increased over the years; they stated that the national guidelines on endometriosis had contributed to increased knowledge.

*I was truly happy when we got national guidelines on endometriosis. It meant that you didn’t need to encounter doctors in the GED who would say, ‘I don’t know anything about your disease, would you tell me about it?’ (1)*

Limited knowledge about endometriosis among HCPs still existed and was one of the reasons why the women experienced playing the odds when seeking GED care. The women felt that more knowledge about endometriosis in general was needed, as well as implementation of care plans in accordance with the national guidelines; this might decrease the feeling of entering the lottery when seeking emergency care.

#### A wish for someone responsible and a plan to be followed

Some of the women expressed a strong need to have someone take responsibility for their care, as they were seeing many different HCPs at many different health care institutions, which was exhausting. They felt that no one took the responsibility to develop a plan for them.

*In the GED, I hear, ‘No, your doctor has to do that.’ Talking with the care center, they say, ‘No,* we *can’t do that.’ (1)*

The women expressed a strong wish for a plan, both for themselves and for the HCPs. A well-developed plan would provide a structured approach to their long-term treatment, encompassing medical, therapeutic, and supportive care. Not only would this provide women with a sense of security and confidence, but it would also lead to better communication and coordination among the HCPs involved in their care.

Only one of the women had a plan, and she expressed that it had importantly changed the care she was receiving.

*We have a care plan. It’s what has been the most decisive thing for me in terms of how I feel in my acute phases. (10)*

The other women said that having a plan would help improve the care they received at the GED. Such a plan would include their special and nursing care needs based on their medical history and ongoing treatments. A plan would reduce anxiety in a stressful situation when seeking GED care.

*Yeah, and it, it’s like… –it’s widely known that there are extreme variations in terms of how endometriosis patients are treated by the health care system. So there’s always some uncertainty as to whether or not you’ll get help when you go in. So a care plan would also end that uncertainty. (2)`*

## Discussion

This qualitative study is, to our knowledge, the first to explore patient experiences and expectations in women with endometriosis in the ED setting. Our findings show that acute and unmanageable pain is the primary cause of women with endometriosis repeatedly seeking GED care. Living with endometriosis was described by all women as having a large impact on their daily life because of endometriosis pain as well as fatigue.

According to the women in our study, multiple aspects of life were influenced by pain, including work or study and relations with friends and family. This caused social, academic, and work disruptions, which have also been described by others in the context of endometriosis [18, 19]. In a qualitative study from New Zealand, where participants were recruited from endometriosis support groups, Huntington and Gilmour (2005) concluded that the lives of women were shaped by chronic pain and that women experienced limitations in different areas of life. A Swedish qualitative study in which women with endometriosis were recruited from a pain clinic described how the women limited, and they struggled because of sick leave and missed education [20]. An Australian study explored women’s experiences of the impact of endometriosis and potential differences across age groups and found that the areas most impacted were marital/sexual relationships, social life, and psychological/physical aspects, regardless of age [21]. Therefore, the consequences and experiences of living with endometriosis were similar to those found in the present study, even though we included only women with endometriosis who repeatedly sought care at the GED.

Many of the women described heightened stress when visiting the GED. This was caused by uncertainty about which HCPs would see them and what level of knowledge these HCPs had regarding their condition. They were unsure whether they would be listened to at all and about what kind of pain treatment they would receive. The women described the process as being like playing the lottery; many felt that a proper plan would minimize their worries when seeking GED care, as the assessment and treatment would then follow a clearly set out plan.

The women therefore described visits to the GED in terms of three interacting factors–the pain; stress about which HCPs would be treating them; and uncertainty regarding the pain treatment–all three having a large impact on the visit. As with the women included in this study, pain has been reported as a common cause for seeking emergency care among patients with other diagnoses [6]. Pain sensitization is a complex process in which the nervous system becomes more sensitive to painful stimuli, which may explain why some individuals experience pain more intensely than others [22]. It`s important to remember that although endometriosis can be a source of pain, any new or atypical pain experience should be carefully evaluated to ensure an accurate diagnosis and appropriate treatment [22–24].

The women in our study described that they sometimes waited too long before seeking emergency care, and one of the main reasons was the uncertainty they felt, as the GED experience felt like playing with chance. The waiting time both at home and at the GED made them feel more stressed and anxious, and often the pain would increase.

Their emergency care encounter depended on the HCPs’ level of knowledge and attitude regarding their diagnosis. In cases where the women met with knowledge and felt acknowledged, they felt comfortable, safe, and less stressed. In contrast, when they saw HCPs with little knowledge regarding their diagnosis and were met with distrust, they felt diminished, unsafe, and stressed. The importance of a good relationship between patients with endometriosis and HCP is highlighted by another study, summarized as respect for patients “values, preferences and needs” and “Information, communication and education” [22], the same values expressed by the women in our study. Other studies have shown that both *acute pain* and *intense pain* can provoke an emotional reaction and that it is important to also assess the patient’s psychological status when treating pain. The waiting times associated with typical ED visits may contribute to patient anxiety and distress, impacting the overall pain experience [15, 25, 26]. The same impact on overall pain experience is likely to be true also at the GED due to unpredictable waiting times, and this corroborates the women’s narratives in our study.

The majority of the women in this study suggested that a health plan would minimize their sense of entering the lottery every time they went to the GED. They felt that such a plan would help reduce their anxiety and stress and possibly also lower the level of pain they experienced when deciding to visit GED. The plan could include a pain management schedule consisting of both medical treatment and alternative analgesia treatment, nursing care advice, and strategies and exercises to ease the pain. Such a plan would make the GED visit more predictable and consequently less stressful. It would also decrease the feeling that the treatment is up to chance, as the individual HCP on call would be of less importance. The women also highlighted the importance of communication between primary care and the hospital; in this respect, the plan could be the connection and improvement of care before and after GED visits. The expressed need for a plan in this study are in line with the European Society of Human Reproduction and Embryology (ESHRE) recommendations for individual plans for women with endometriosis, as these standardize the treatment and make it predictable [1, 27]. The PCC approach helps patients feel more empowered in managing their illness and increases their confidence [28–30].

Our study has shown the importance of patients feeling listened to and acknowledged by the HCP at the GED, which is in agreement with other studies investigating what is of great value for patients with other diagnoses at the ED [26]. The experiences and expectations of the women participating in our study support the shift towards person-centred care (PCC). PCC is based on the patient’s own story, conditions, and resources, where the patient’s obstacles are taken into account [31]. A Swedish study showed that having a responsible gynaecologist was an independent predictor for high patient-centeredness as being the responsible gynaecologist includes making a health plan follow-up [30]. The findings from another study indicate that patient-centredness leads to better quality of life [32]. By incorporating PCC into the care of women with endometriosis, HCP´s can better understand their unique experiences and expectations. This approach may contribute to a more holistic and personalized plan, ultimately improving the overall quality of care and patient outcomes. More effort to develop the relational aspects of care seems to be a way for better care, it builds a picture with more knowledge of women´s perspective were PCC could be a tool to evolve, which our and other studies indicate [30, 32, 33].

This study focused on women’s experiences of living with endometriosis and their experience of frequently having to visit the GED. A qualitative method was used to identify and describe the women’s experiences. This method provided detailed data on essential aspects from the women, which could not have been captured through a quantitative survey. The study population of ten women may seem limited; however, the interviews generated a large amount of rich and informative data, which was sufficient to achieve both depth and width in the analysis.

To establish trustworthiness in this study, all steps of the data collection and analysis process have been described, and quotations have been used to illustrate the themes and verify that the findings are grounded in the women’s narratives. To avoid overinterpretation, the research team analysed the data separately, discussed the analysis, and reached agreement in the interpretation. The results of qualitative research are not intended for generalization, but the results of this study might be transferable to other women with endometriosis in similar contexts.

This study contributes to the limited knowledge about women with a diagnosis of endometriosis who frequently seek emergency care. To our knowledge, this is the first study with the specific aim of investigating their perspectives and experiences. The interviews provided rich material, where similar themes emerged between the patients.

In addition to the new knowledge regarding women with endometriosis and repeat ED use, this study also adds valuable information on the GED setting, where very few studies have been performed. As little is known about patients’ experience in the GED setting, this study also contributes to the development of care in this field.

The study also has some limitations. Both the interviewers were registered nurses, and although none worked at the GED, one worked at the gynaecological clinic, but had had no interactions with the included patients. The patients were informed about the interviewers’ backgrounds, and being interviewed by an HCP could be seen as a power imbalance and affect the willingness to share experiences. On the other hand, this could also have favoured trust and openness during the interviews. Our impression was that all women honestly shared their experiences during the interviews. Second, two of the interviews were performed digitally, and this could have influenced the interaction and sharing experiences; however, this was according to the participants choice. Third, the study did not include persons who did not speak Swedish. This limitation could affect the transferability of the findings, as the ED treats patients with different ethnic backgrounds. Finally, the interviews were conducted at one GED, and the experiences may differ at other clinics.

### Knowledge gaps

To our knowledge, this is the first study carried out on patients’ experiences and expectations in the context of living with endometriosis and repeated GED visits, and our findings need to be explored further. The benefits of a health plan in the context of endometriosis should be studied in future research. In addition, we have identified a lack of studies regarding endometriosis and PCC, an additional important knowledge gap in this large patient group.

To further develop care at the GED, investigating HCPs’ experiences and expectations of treating and caring for women with endometriosis at the GED is needed. As one of the interviewed women said,

*Thank you so much for taking the time and doing what you’re doing here because it’s so important and a step in the right direction. We must never be silenced again. For our own sake, and for the sake of our children and our grandchildren moving forward, like. (2)*

## Conclusions and implications

Women with endometriosis who repeatedly need to use the GED still mostly lack individualized health plans. Pain was the reason why the women we interviewed needed to visit the GED, but many of the women felt uncertainty before the visit, as they did not know what kind of treatment they would receive, and many expressed that the most important thing for them was to be listened to. This study contributes to the limited research on women’s experiences of living with endometriosis combined with experiences of GED visits. Our findings support the development of PCC.

### Relevance to clinical practice

Our findings have identified several areas where the care of women with endometriosis can be improved. We have shown that multiple visits to the GED in women with endometriosis are due to pain, indicating that pain management should be prioritized. Many women experienced that a visit at the GED could be compared to entering a lottery, as they did not know what kind of treatment they would receive, and this was associated with negative feelings including uncertainty. A health plan could improve this problem, while simultaneously facilitating the assessment and treatment at the GED.

## Supporting information

S1 File Interview guide–frequent GED users. (PDF)
