# Supplementary material for: Endometriosis leading to frequent emergency department visits–women’s experiences and perspectives
Source: PLoS One. 2024 Nov 21;19(11):e0307680. doi: 10.1371/journal.pone.0307680 (PMC11581228; doi:10.1371/journal.pone.0307680)
Supplement: S1 File — (PDF) [file pone.0307680.s001.pdf]

INTERVIEW GUIDE – Frequent GED Users

The purpose of the interview is to conduct a conversation using open-ended questions with women diagnosed with endometriosis who sought emergency care at a gynecological emergency department (GED) on at least four occasions in 2021. The conversation includes two topics:

1. Perceptions and experiences regarding endometriosis
2. Perceptions and experiences regarding nursing and care in connection with endometriosis

Is there anything you are wondering about before we begin?

1. Perceptions and experiences regarding endometriosis

Introductory question:
Can you freely describe your experiences of living with endometriosis?

Support questions:
- How is endometriosis affecting your life?
- If you were to explain your condition to someone who does not know anything about having endometriosis, what would you tell them?
- What do you see as the worst thing about endometriosis?
- What would your life look like if you did not have endometriosis?

2. Perceptions and experiences regarding nursing and care in connection with endometriosis

Introductory question:
Can you tell me about your experiences of health care in connection with endometriosis?

Support questions:
- How could the care given for endometriosis be improved?
- What expectations did you have in connection with the GED visits?
- What, do you think, could help you avoid seeking emergency care?
- Is there any particular part of the care that would need to be improved in order for it to work better for you? How would that work?
- Is there any part of the nursing/care that you think works well? What is working well?

Concluding questions:
- Do you have anything to add beyond what we talked about?
- What, do you think, is the most important thing we talked about?

Patient data:
- How old are you?
- Do you live alone or with family?
- Where were you born? How many years have you lived in Sweden?
- How many years of schooling do you have?
- What is your profession?
- What is your primary occupation right now?
  (working, studies, seeking work, other)
- What health care services are you in contact with for your endometriosis symptoms at present?
  (gynecology at the hospital, outpatient gynecology, care center, psychiatry, physiotherapy)
- How long have you had endometriosis?

Thanks for the chat!
